# Supplementary material for: Neurofibromin 1 controls metabolic balance and Notch-dependent quiescence of murine juvenile myogenic progenitors
Source: Nat Commun. 2024 Feb 15;15:1393. doi: 10.1038/s41467-024-45618-z (PMC10869796; doi:10.1038/s41467-024-45618-z)
Supplement: Supplementary file 1 — Supplementary Information [file 41467_2024_45618_MOESM1_ESM.pdf]

Supplementary Figures and Tables

Neurofibromin 1 controls metabolic balance and Notch-dependent quiescence of murine juvenile myogenic progenitors

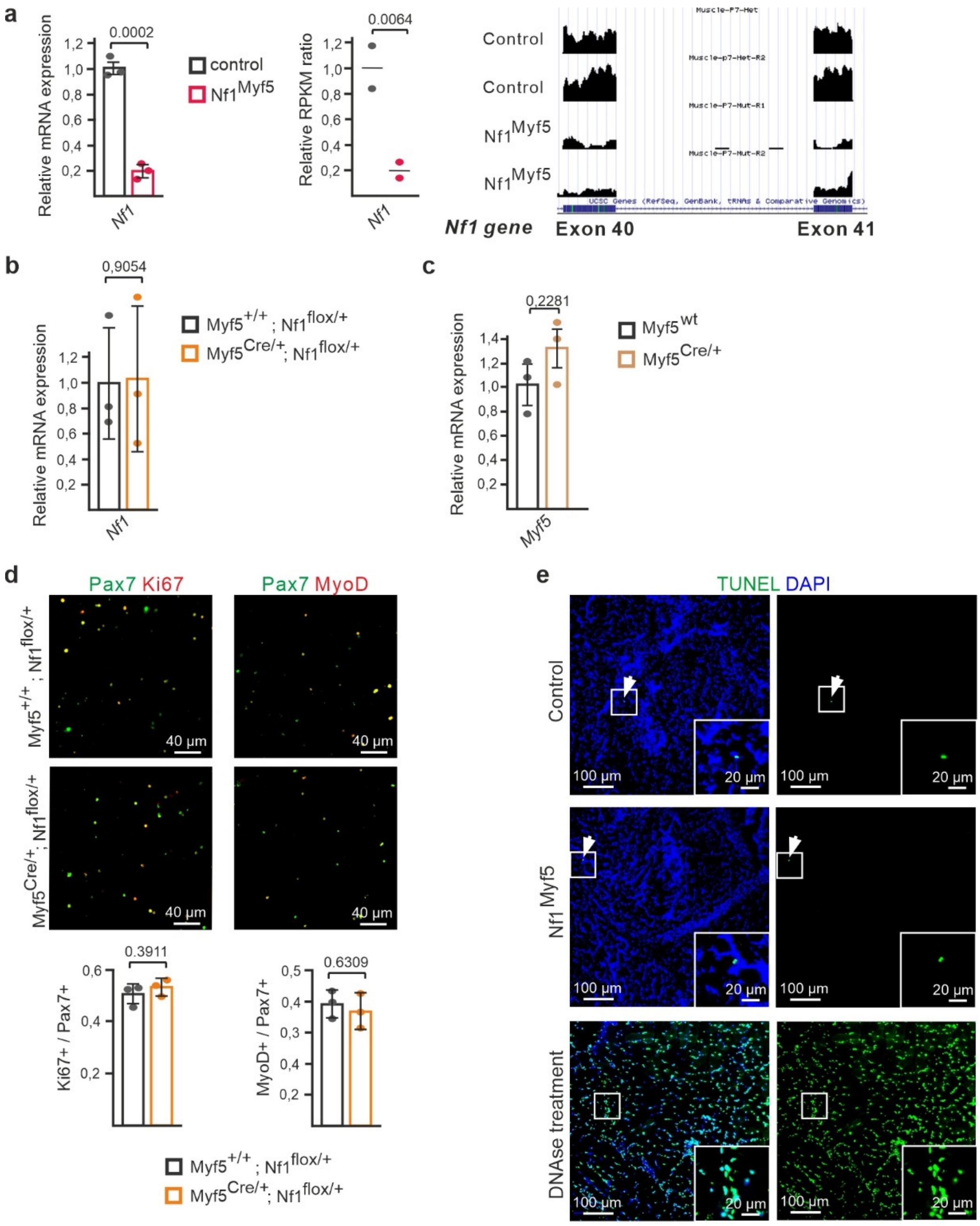

Supplementary Fig. 1. Knockdown efficiency of *Nf1*; analysis of Nf1 haploinsufficient mice; apoptosis analysis in Nf1^{Myf5} mice

a) Analysis of Nf1 knockdown efficiency in FACS isolated p7 MPs from control and Nf1^{Myf5} mice by RT-qPCR (left; n=3 animals per genotype; p-value shown) and mRNA-Sequencing (right, 2 animals per genotype; mean values and Padj.-value shown). **b)** Analysis of *Nf1* expression in MPs of *Nf1* control (Myf5$^{+/+}$;Nf1$^{flox/+}$) or haploinsufficient (Myf5^{Cre};Nf1$^{flox/+}$) animals (n=3 animals per genotype; p-value shown). **c)** Analysis of *Myf5* expression in p7 MPs from Myf5^{wt} vs. Myf5$^{Cre/+}$ animals (n=3 animals per genotype; p-value shown). **d)** Cytospin of FACS-isolated p14 MPs from control or *Nf1* haploinsufficient (Myf5^{Cre};Nf1$^{flox/+}$) mice labeled for Pax7 (green) and Ki67 (red) or Pax7 (green) and MyoD (red), respectively; quantifications shown below (n = 3 animals per genotype; p-values shown). **e)** Analysis of apoptosis assessed by TUNEL staining (green) on p14 muscle sections. In both, controls as well as Nf1^{Myf5} muscle, only occasional TUNEL+ cells can be seen. Right panel: positive control that was treated with DNAse. Data are mean ± SEM; P-value calculated by two-sided unpaired t-test. Source data are provided as a Source Data file.

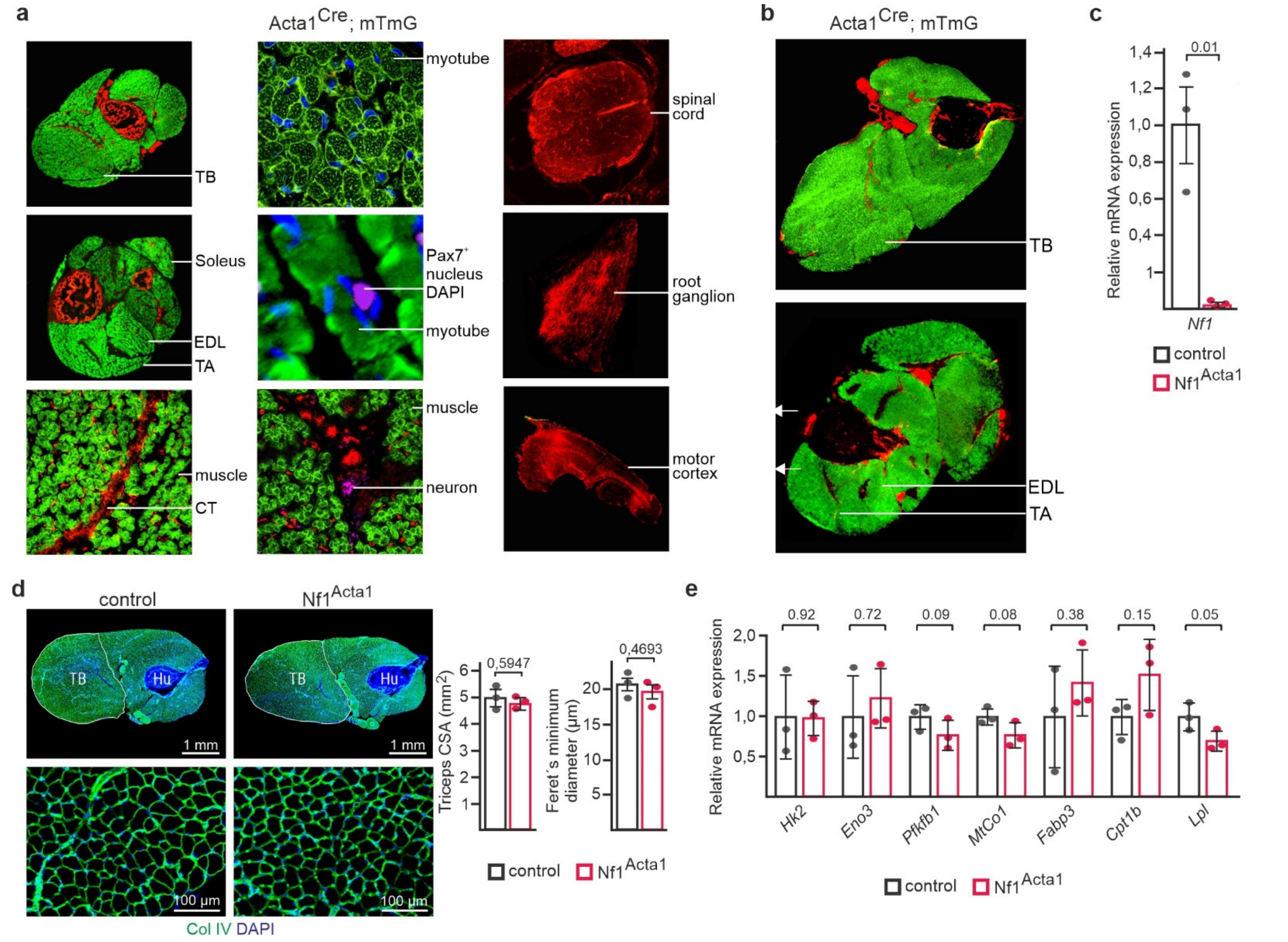

Supplementary Fig. 2. Analysis of Acta1^{Cre} efficacy; Nf1^{Acta1} phenotype

a, b) Acta1-Cre mice were bred to Rosa26^{mTmG} reporter mice and analyzed at embryonic day 18 (a) or postnatal day 21 (b). Green depicts mG reporter activity (recombination), red depicts mT reporter activity (no recombination). Satellite cells have been stained for Pax7 (purple), neurons for β-tubulin III (purple). **c)** RT-qPCR analysis of *Nf1* deletion efficacy in Nf1^{Acta1} postnatal day 21 muscle tissue (n=3 animals per genotype; p-value shown). **d)** Cross sections of upper forelimbs of control and Nf1^{Acta1} mice at p21 stained for Laminin (green) and DAPI (blue; nuclei). TB, triceps brachii; Hu, humerus. Magnification from TB muscle shown below. Right: Quantification of TB cross-sectional area (left) and myofiber Feret's minimum diameter (right) of control and Nf1^{Acta1} TB muscle (n = 3 animals per genotype; p-values shown). **e)** RT-qPCR analysis of indicated metabolic genes in control and Nf1^{Acta1} p21 muscle tissue (n = 3 animals per genotype; p-values shown). Data are mean ± SEM; P-value calculated by two-sided unpaired t-test. Source data are provided as a Source Data file.

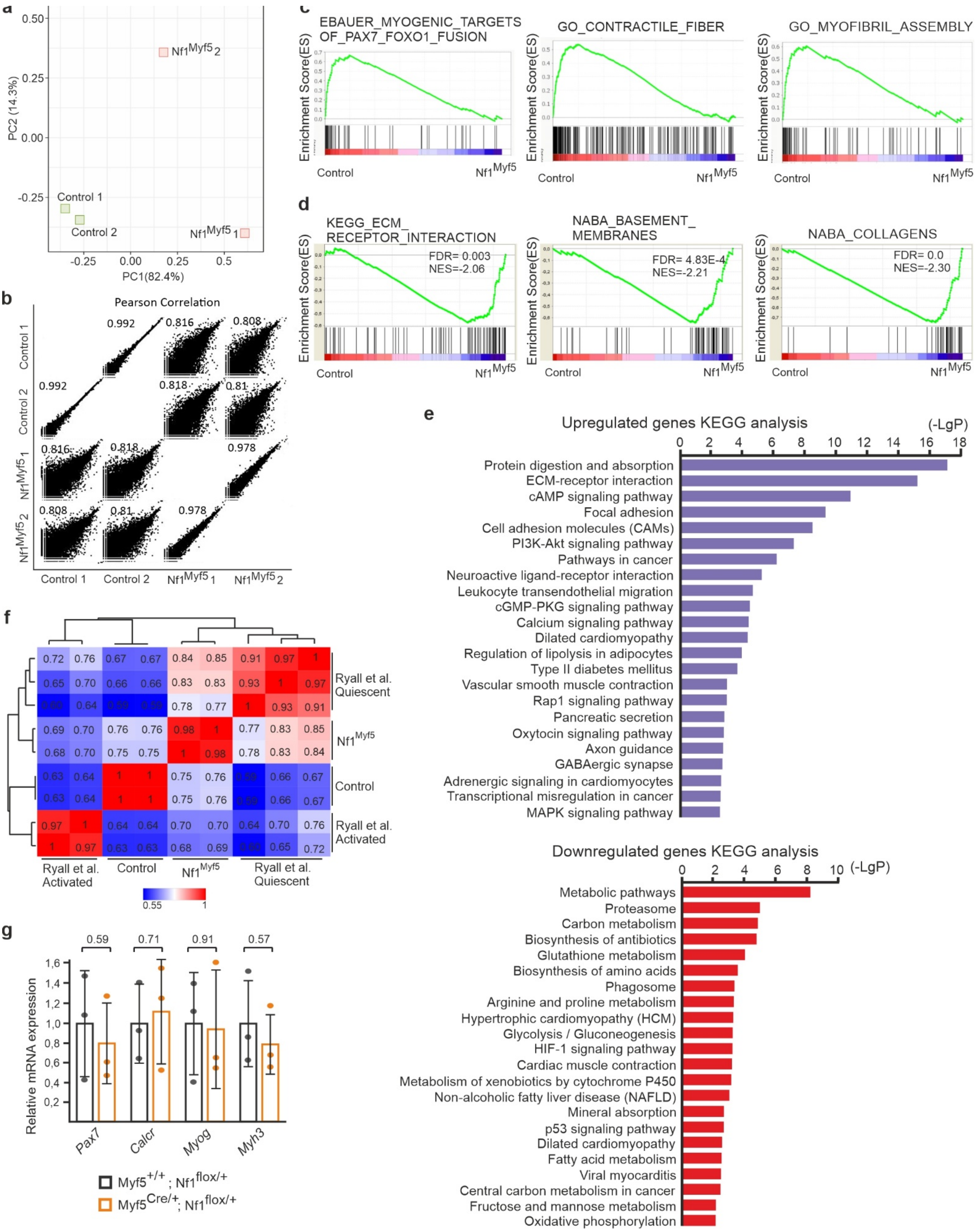
a
PC2 (14.3%)
PC1(82.4%)
Control 1
Control 2
Nf1Myf5 1
Nf1Myf5 2
b
Pearson Correlation
Control 1
Control 2
Nf1Myf5 1
Nf1Myf5 2
0.992
0.816
0.808
0.818
0.81
0.978
c
EBAUER_MYOGENIC_TARGETS OF_PAX7_FOXO1_FUSION
GO_CONTRACTILE_FIBER
GO_MYOFIBRIL_ASSEMBLY
Enrichment Score(ES)
Control
Nf1Myf5
d
KEGG_ECM_ RECEPTOR_INTERACTION
FDR= 0.003
NES=-2.06
NABA_BASEMENT_ MEMBRANES
FDR= 4.83E-4
NES=-2.21
NABA_COLLAGENS
FDR= 0.0
NES=-2.30
e
Upregulated genes KEGG analysis
(-LgP)
Protein digestion and absorption
ECM-receptor interaction
cAMP signaling pathway
Focal adhesion
Cell adhesion molecules (CAMs)
PI3K-Akt signaling pathway
Pathways in cancer
Neuroactive ligand-receptor interaction
Leukocyte transendothelial migration
cGMP-PKG signaling pathway
Calcium signaling pathway
Dilated cardiomyopathy
Regulation of lipolysis in adipocytes
Type II diabetes mellitus
Vascular smooth muscle contraction
Rap1 signaling pathway
Pancreatic secretion
Oxytocin signaling pathway
Axon guidance
GABAergic synapse
Adrenergic signaling in cardiomyocytes
Transcriptional misregulation in cancer
MAPK signaling pathway
Downregulated genes KEGG analysis
(-LgP)
Metabolic pathways
Proteasome
Carbon metabolism
Biosynthesis of antibiotics
Glutathione metabolism
Biosynthesis of amino acids
Phagosome
Arginine and proline metabolism
Hypertrophic cardiomyopathy (HCM)
Glycolysis / Gluconeogenesis
HIF-1 signaling pathway
Cardiac muscle contraction
Metabolism of xenobiotics by cytochrome P450
Non-alcoholic fatty liver disease (NAFLD)
Mineral absorption
p53 signaling pathway
Dilated cardiomyopathy
Fatty acid metabolism
Viral myocarditis
Central carbon metabolism in cancer
Fructose and mannose metabolism
Oxidative phosphorylation
f
Ryall et al. Quiescent
Nf1Myf5
Control
Ryall et al. Activated
0.55
1
g
Relative mRNA expression
0.59
0.71
0.91
0.57
Pax7
Calcr
Myog
Myh3
Myf5+/+; Nf1flox/+
Myf5Cre/+; Nf1flox/+

Supplementary Fig. 3. RNA-Seq analysis of p7 Nf1^{Myf5} MPs

a) Principal component analysis of mRNA-Sequencing data of control and Nf1^{Myf5} p7 MPs. **b)** Pearson correlation analysis between mRNA-Sequencing samples **c, d)** GSEA of control and Nf1^{Myf5} p7 MP RNA-Seq data for "Ebauer myogenic targets of Pax7 Foxo1 fusion", "GO contractile fiber", "GO myofibril assembly"; and "ECM Receptor Interaction", "Basement Membranes" and "NABA Collagens". **e)** KEGG analysis of genes downregulated (upper panel) or upregulated (lower panel) in Nf1^{Myf5} p7 MPs vs. control MPs. **f)** Correlation analysis of transcriptome data from Ryall et al. (2015) and transcriptomes of control or Nf1^{Myf5} p7 MPs. **g)** Analysis of *Pax7*, *Calcr*, *Myog* and *Myh3* expression in MPs of Nf1 control or haploinsufficient (Myf5^{Cre};Nf1$^{flox/+}$) animals (n=3 animals per genotype; p-value shown). Data are mean ± SEM; P-value calculated by two-sided unpaired t-test. Source data are provided as a Source Data file.

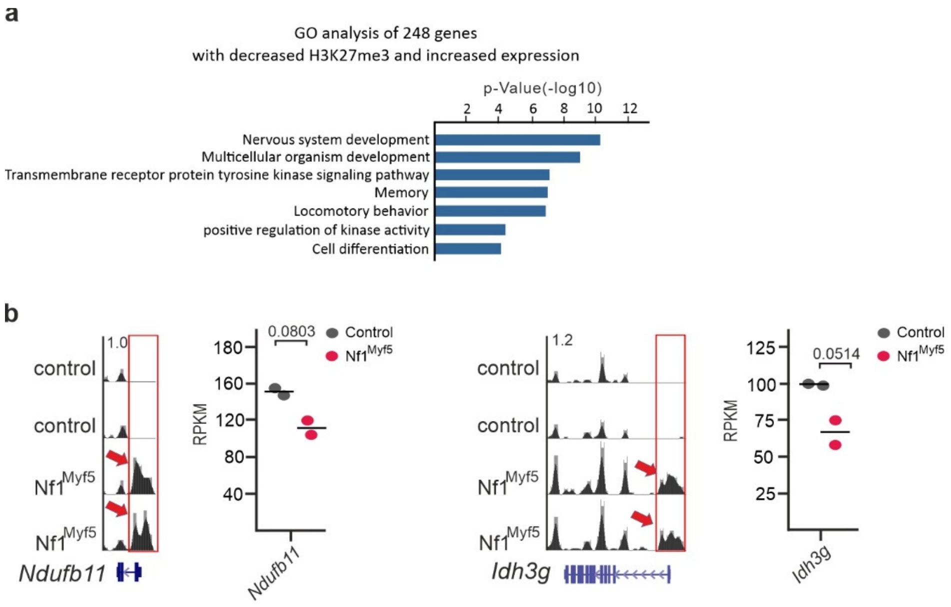

Supplementary Fig. 4. H3K27me3 and DNA methylation analysis in Nf1^{Myf5} MPs

a) GO analysis of 248 genes with reduced H3K27me3 and downregulated mRNA expression in p7 Nf1^{Myf5} MPs (see Venn diagram in main Figure 4e). **b)** MeDIP-Seq tracks from control and Nf1^{Myf5} p7 MPs at the *Ndufb11* and *Idh3g* loci. RPKM values for *Ndufb1* and *Idh3g* expression levels from RNA-Seq of p7 MPs is shown to the right (n = 2; mean values and Padj.-values are shown). Source data are provided as a Source Data file.

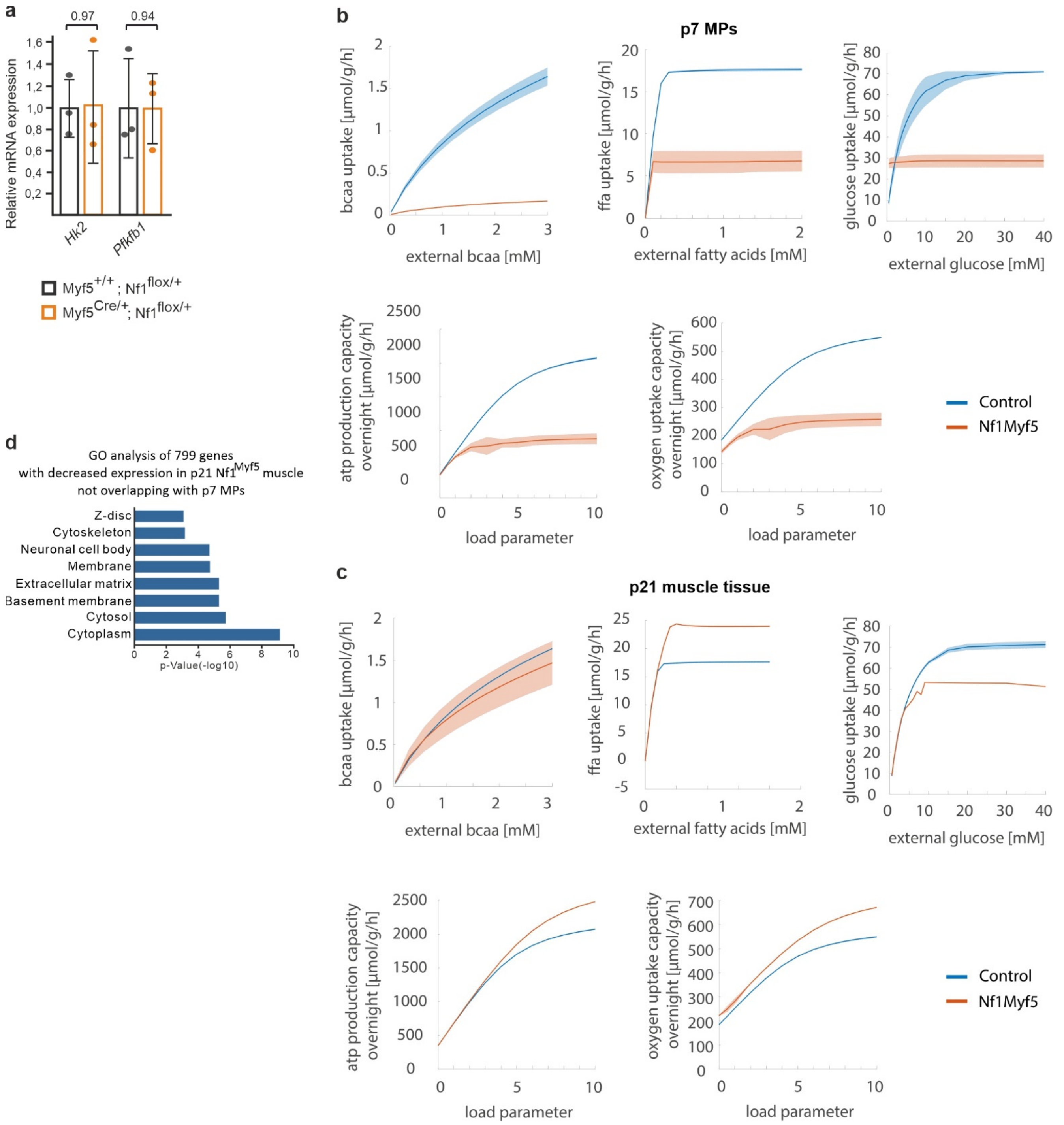

Supplementary Fig. 5 Metabolic analysis of Nf1-deficient MPs and muscle

a) RT-qPCR analysis of *Hk2* and *Pfkfb1* expression in p7 MPs of control or haploinsufficient (Myf5^{Cre};Nf1$^{flox/+}$) animals (n = 3 animals per genotype; p-values shown). **b, c)** Kinetic metabolic flux modeling on transcriptome data of control and Nf1^{Myf5} p7 MPs (b) and p21 muscle tissue (c). Lines depict mean, colored area depicts standard deviation. **d)** GO analysis of 799 genes downregulated in p21 Nf1^{Myf5} muscle, but not in p7 Nf1^{Myf5} MPs (see Venn diagram in Fig. 5g). Data in (a) are mean ± SEM; P-value calculated by two-sided unpaired t-test. Source data are provided as a Source Data file.

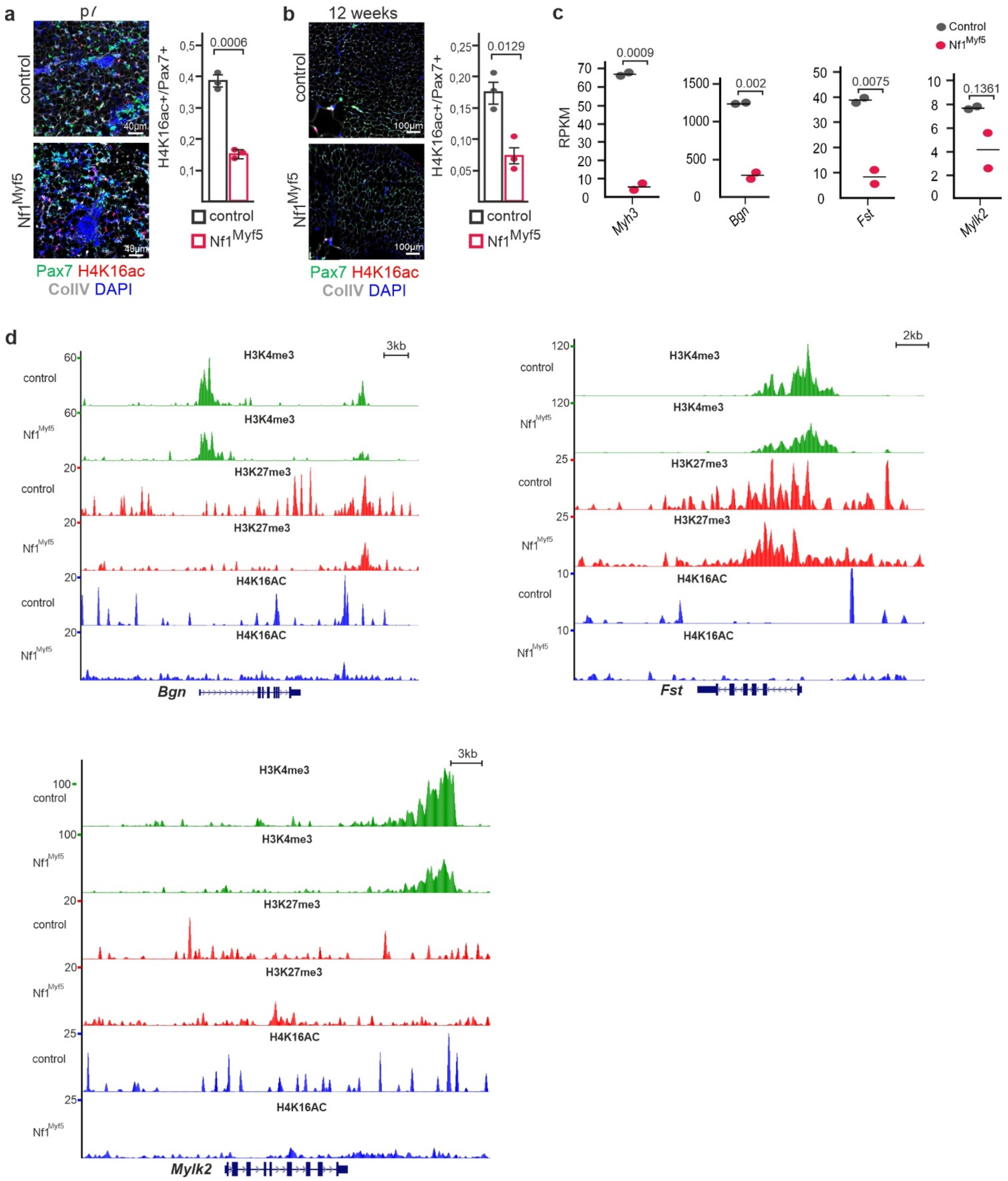

Supplementary Fig. 6. Analysis of H4K16ac in Nf1^{Myf5} mice

a, b) Immunolabeling for Pax7 (green) and H4K16ac (red) on sections of TA muscles from p7 (a) and 12 weeks old (b) control and Nf1^{Myf5} animals; quantification of H4K16ac+/Pax7+ cells relative to all Pax7+ cells is shown right (n=3 animals per genotype; p-values shown). **c)** RPKM values from RNA-Seq data of control and Nf1^{Myf5} p7 MPs for *Bgn, Fst* and *Mylk2* (n = 2 animals; mean values and Padj.-values shown). **d)** H3K4me3, H3K27me3, H4K16ac ChIP-Seq tracks from control and Nf1^{Myf5} p7 MPs at the *Bgn, Fst* and *Mylk2* loci. Source data are provided as a Source Data file.

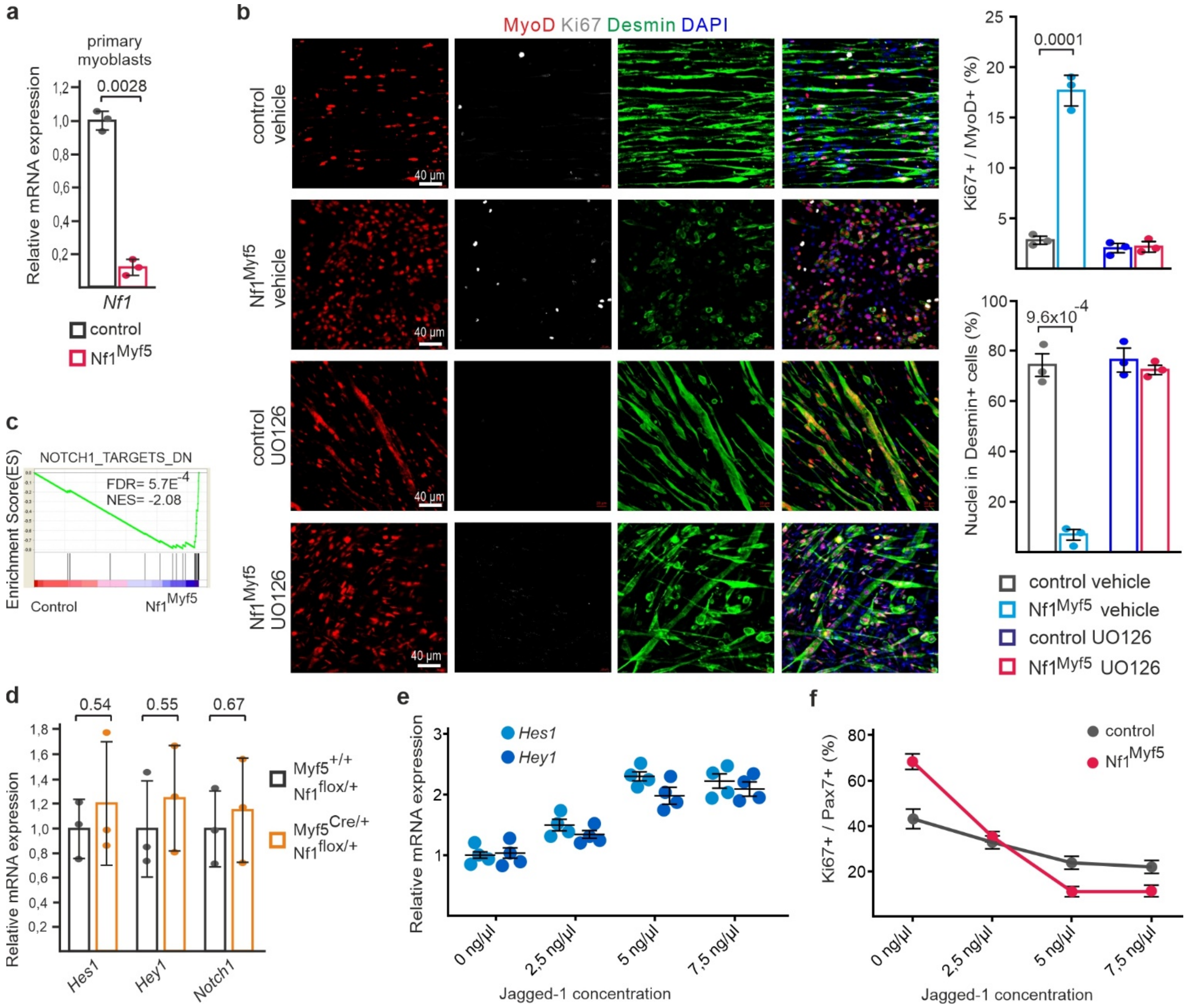

Supplementary Fig. 7. Analysis of Nf1^{Myf5} primary myoblasts

a) RT-qPCR analysis of *Nf1* expression in primary myoblasts from control and Nf1^{Myf5} mice (n = 3 animals per genotype; p-value shown). **b)** Culture of control and Nf1^{Myf5} primary myoblasts immunolabeled for MyoD (red), Ki67 (grey), Desmin (green) and DAPI (blue; nuclei). Cells were cultured in differentiation medium without addition, or with addition of MEK inhibitor UO126 for 2 days. Quantification of Ki67+ nuclei amongst MyoD+ nuclei, and quantification of nuclei in Desmin+ cells are shown right (3 independent experiments from n = 3 animals per genotype; p-values shown). **c)** GSEA on RNA-Seq data from control and Nf1^{Myf5} p7 MPs for "Notch targets". **d)** Analysis of *Hes1*, *Hey1* and *Notch1* expression in MPs of Nf1 control or haploinsufficient (Myf5^{Cre};Nf1$^{flox/+}$) animals (n=3 animals per genotype; p-values shown). **e)** Dose-response curve of indicated Jagged-1 ligand concentrations on Notch target gene (*Hes1*, *Hey1*) expression in wild type MPs. Expression levels in untreated MPs were set as 1 (n = 3 independent experiments from n = 3 animals). **f)** Dose-response curve of indicated Jagged-1 ligand concentrations on control and Nf1^{Myf5} MP proliferation measured as numbers of Ki67+/Pax7+ cells (n = 3 animals). Data are mean± SEM; P-value calculated by two-sided unpaired t-test. Source data are provided as a Source Data file.

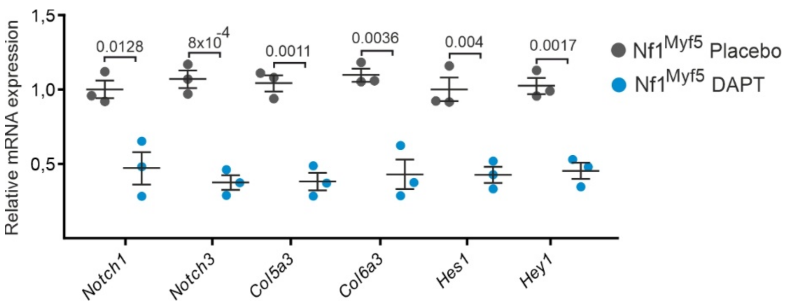

Supplementary Fig. 8. DAPT treatment of Nf1^{Myf5} animals

RT-qPCR analysis of Notch pathway target gene expression in p21 TA muscle from Nf1^{Myf5} mice treated with placebo or DAPT (n= 3 animals for each condition; each dot represents the mean value of three replicates from one sample; p-values shown). Data are mean ± SEM; P-value calculated by two-sided unpaired *t*-test. Source data are provided as a Source Data file.

Supplementary Table 1. Antibodies

Antibody	Source	Identifier
**Primary antibodies:**		
Goat anti-Collagen IV	Millipore	Cat# AB769; RRID: AB_92262
Goat anti-Desmin	R&D Systems	Cat# AF3844; RRID: AB_2092419
Mouse anti-Pax7	DSHB	Cat# pax7, RRID: AB_528428
Guinea pig anti-Pax7	Carmen Birchmeier, Max Delbrück Center for Molecular Medicine Berlin, cbirch@mdc-berlin.de	N/A
Rabbit anti-Ki67	Abcam	Cat# ab16667, RRID: AB_302459
Mouse anti-Ki67	BD Biosciences	Cat# 550609, RRID: AB_393778
Rabbit anti-MyoD	Cell Signaling Technology	Cat# 13812, RRID: AB_2798320
Mouse anti-MyoD	BD Biosciences	Cat# 554130, RRID: AB_395255
Mouse anti-MF20	DSHB	Cat# MF 20, RRID: AB_2147781
Anti-Myosin (Skeletal, Fast) antibody	Sigma-Aldrich	Cat# M1570, RRID: AB__2147168
Mouse anti-MyHC type 1	DSHB	Cat# BA-D5, RRID: AB_2235587
Mouse anti-MyHC type 2A	DSHB	Cat# SC-71, RRID: AB_2147165
Mouse anti-MyHC type 2B	DSHB	Cat# BF-F3, RRID: AB_2266724
Rabbit anti-phospho (Thr 389)-p70s6k	Cell Signaling Technology	Cat# 9205, RRID: AB_330944
Rabbit anti-p70s6k	Cell Signaling Technology	Cat# 9202, RRID: AB_331676
Rabbit anti- pERK1/2	Cell Signaling Technology	Cat# 9101, RRID: AB_331646
Phospho-S6 Ribosomal Protein (Ser235/236)	Cell Signaling Technology	Cat# 4858, RRID: AB_916156
Anti-acetyl-Histone H4 (Lys16)	Millipore	Cat# 07-329, RRID: AB_310525
Anti-trimethyl-Histone H3 (Lys4)	Millipore	Cat# 07-473, RRID: AB_1977252
Anti-trimethyl-Histone H3 (Lys27)	Millipore	Cat# 07-449, RRID: AB_310624
Mouse anti-β-Tubulin III	Sigma-Aldrich	Cat# T8578, RRID: AB_1841228
**Secondary Antibodies:**		
Alexa Fluor 488 Donkey anti-Mouse	Thermo Fisher	Cat# A-21202, RRID: AB_141607
Alexa Fluor 568 Donkey anti-Mouse	Thermo Fisher	Cat# A10037, RRID: AB_2534013
Alexa Fluor 488 Donkey anti-Rabbit	Thermo Fisher	Cat# A-21206, RRID: AB_141708
Alexa Fluor 568 Donkey anti-Rabbit	Thermo Fisher	Cat# A10042, RRID: AB_2534017
Alexa Fluor 488 Donkey anti-Goat	Thermo Fisher	Cat#A-11055, RRID: AB_2534102
Alexa Fluor 568 Donkey anti-Goat	Thermo Fisher	Cat# A-11057, RRID: AB_2534104
Alexa Fluor 680 Donkey anti-Goat	Thermo Fisher	Cat# A-21084, RRID: AB_2535741
Alexa Fluor 680 Goat anti-Guinea pig	Thermo Fisher	Cat# SA5-10098, RRID: AB_2556678
Alexa Fluor 647 Goat anti-Mouse IgG1	Thermo Fisher	Cat# A-21240, RRID: AB_2535809
Alexa Fluor 488 Goat anti-Mouse IgM (Heavy chain)	Thermo Fisher	Cat# A-21042, RRID: AB_2535711
Alexa Fluor 555 Goat anti-Mouse IgG2b	Thermo Fisher	Cat# A-21147, RRID: AB_2535783
HRP Goat anti-Rabbit	Thermo Fisher	Cat# A27036, RRID: AB_2536099
HRP Goat anti-Mouse	Thermo Fisher	Cat# G-21040, RRID: AB_2536527
**FACS antibodies:**		
Rat anti-CD31(PECAM1) APC	Thermo Fisher	Cat# 17-0311-82, RRID: AB_657735
Rat anti-CD45 APC	Thermo Fisher	Cat# 17-0451-83, RRID: AB_469393
Rat anti-Ter119 APC	Thermo Fisher	Cat# 17-5921-83, RRID: AB_469474
Rat anti-Ly-6A/E (Sca1) APC-Cy7	BioLegend	Cat# 108126, RRID: AB_10645327
Rat anti-Integrin alpha 7 PE	R&D Systems	Cat# FAB3518P, RRID: AB_2128441

Supplementary Table 2. Primer Sequences (RT-qPCR)

Nf1_fw	ACAAAGGGTTACTGCCAT
Nf1_rev	GCTCCCCCAGATTTTTGC
Myf5_fw	TGAGGGAACAGGTGGAGAAC
Myf5_rev	CTGTTCTTTCGGGACCAGAC
Pax7_fw	CCGTGTTTCTCATGGTTGTG
Pax7_rev	GAGCACTCGGCTAATCGAAC
Spry1_fw	TAGGTCAGATCGGGTCATCC
Spry1_rev	TTCGCAGATGAACTTGTGCT
Notch1_fw	AGGCAAATGCCTCAACACAC
Notch1_rev	CATTGGAACTCCCCAATCTG
Notch3_fw	AGGGCCAGAACTGTGAAGTC
Notch3_rev	AGGGCACTGGCAGTTGTAAG
Hes1_fw	TCATCAAAGCCTATCATGGAGA
Hes1_rev	AGGTGCTTCACAGTCATTTCC
Hey1_fw	CCGACGAGACCGAATCAATA
Hey1_rev	TTTTCAGGTGATCCACAGTCA
Myod1_fw	AGCACTACAGTGGCGACTCA
Myod1_rev	GCTCCACTATGCTGGACAGG
MyoG_fw	CTACAGGCCTTGCTCAGCTC
MyoG_rev	AGATTGTGGGCGTCTGTAGG
Myh3_fw	GGACGCTGGAGGATCAAAT
Myh3_rev	AAAATGGATGCGGATGAACT
ATP2a1_fw	CTGACCGAAAGTCAGTGCAA
ATP2a1_rev	GGTGGATTTGATGGAGAGGA
DNMT1_fw	GAACCCCAGATGTTGACCAG
DNMT1_rev	GGTGTCGACAGGACACAGGT
DNMT3a_fw	ACTTGGAGAAGCGGAGTGAA
DNMT3a_rev	TTCTGGTGGGGTCTCAGTTC
Myl1_fw	ACAACAAGGACCAGGGAGGT
Myl1_rev	CTGCCAGCAACGCTTCTAC
Pfkfb1_fw	TGATGCCACCAACACTACCA
Pfkfb1_rev	CTGCAATGATGTCTGGGTCA
Calcr_fw	AGCCCAACTCCAGTTCTTCA
Calcr_rev	TCCTTCATAAGAGGGCAACTG
Pfkfb3_fw	GCAGTACAGCTCTTACAACTTCT
Pfkfb3_rev	TGTCCACCTTCCTTTGTCAG
Pfkm_fw	ACGTGACCAAGGCTATGGAT
Pfkm_rev	GACTGGGGGTCTGACATGAG
Eno3_fw	AAATCTTCGCCCGGGAAATC
Eno3_rev	TGCTTCATAGATACCCGTGGA
Ldha_fw	ACTTGGCGGATGAGCTTG
Ldha_rev	GCGGTGATAATGACCAGCTT
Hk2_fw	GTTTGACCACATTGCCGAAT
Hk2_rev	CACGCCACTGGACTTGAAC
Ndufv1_fw	CCA TTC TAA TCG CCATAG CC
Ndufv1_rev	GCC GTA TGG ACC AAC AAT G
Mtco1_fw	TGCTAGCCGCAGGCATTAC
Mtco1_rev	GGGTGCCCAAAGAATCAGAAC
Myh1_fw	CGGGAAGACTGTGAACACGA
Myh1_rev	CGTTCCCAAAGGCCTCCA
Myh2_fw	ACCCTCCCAAGTACGACAAG
Myh2_rev	TACACCGGCAGCCATTTGTA
Myh4_fw	CAGAGTCACCTTCCAGCTCA
Myh4_rev	TGATTTCACCTTGACTGACGT
Myh7_fw	AGCTGGGAAGACTGTCAACA
Myh7_rev	CCAAAGGCCTCCAGAGCA
Col5a3_fw	CCTGGGGTGTGCATAGAGAC
Col5a3_rev	GGACACTGAGAAGGCTGGAC
Col6a3_fw	GTGGCTCAGTATGCAGACACT
Col6a3_rev	GTCCAGAGAAGATCCCGTGT
Gapdh-fw	AACTTTGGCATTGTGGAAGG
Gapdh-rev	CAGTCTTCTGGGTGGCAGTG
Actb-fw	CGTGAAAAGATGACCCAGATCA
Actb-rev	GGGACAGCACAGCCTGGAT

Supplementary Table 3. Primer sequences (ChIP-Sequencing; Nextera custom primers)

Ad1noMX	AATGATACGGCGACCACCGAGATCTACACTCGTCGGCAG
Ad2.1_TAAGGCGA	CAAGCAGAAGACGGCATACGAGATTCGCCTTAGTCTCGT
Ad2.2_CGTACTAG	CAAGCAGAAGACGGCATACGAGATCTAGTACGGTCTCGT
Ad2.3_AGGCAGAA	CAAGCAGAAGACGGCATACGAGATTTCTGCCTGTCTCGT
Ad2.4TCCTGAGC	CAAGCAGAAGACGGCATACGAGATGCTCAGGAGTCTCGT
Ad2.5GGACTCCT	CAAGCAGAAGACGGCATACGAGATAGGAGTCCGTCTCGT
Ad2.6_TAGGCATG	CAAGCAGAAGACGGCATACGAGATCATGCCTAGTCTCGT
Ad2.7_CTCTCTAC	CAAGCAGAAGACGGCATACGAGATGTAGAGAGGTCTCGT
Ad2.8CAGAGAGG	CAAGCAGAAGACGGCATACGAGATCCTCTCTGGTCTCGT
Ad2.9_GCTACGCT	CAAGCAGAAGACGGCATACGAGATAGCGTAGCGTCTCGT
Ad2.10_CGAGGCTG	CAAGCAGAAGACGGCATACGAGATCAGCCTCGGTCTCGT
Ad2.11_AAGAGGCA	CAAGCAGAAGACGGCATACGAGATTGCCTCTTGTCTCGT
Ad2.12_GTAGAGGA	CAAGCAGAAGACGGCATACGAGATTCCTCTACGTCTCGT

Supplementary Table 4. Primer sequences (MeDIP Library preparation)

TRUSEQ_UNI_TRUNC	ACACTCTTTCCCTACACGACGCTCTTCCGATC*T
Index_SCAFFOLD	5P-GATCGGAAGAGCACACGTCTGAACTCCAGTCAC
Index1	CAAGCAGAAGACGGCATACGAGATCGTGATGTGACTGGAGTTCAGACGTGTGCTCTTCCGATC
Index8	CAAGCAGAAGACGGCATACGAGATTCAAGTGTGACTGGAGTTCAGACGTGTGCTCTTCCGATC
Index10	CAAGCAGAAGACGGCATACGAGATAAGCTAGTGACTGGAGTTCAGACGTGTGCTCTTCCGATC
Index11	CAAGCAGAAGACGGCATACGAGATGTAGCCGTGACTGGAGTTCAGACGTGTGCTCTTCCGATC
2nd primer	AATGATACGGCGACCACCGAGATCTACACTCTTTCCCTACACGACGCTCTTCCGATCT
1CpG_qPCR_F	ACAAGTTGTTTGATCTTTGC
1CpG_qPCR_R	CCTATGAGCAACGTGTTAG
5CpG_qPCR_F	CACTTGAATCTGTGGTTCAT
5CpG_qPCR_R	TAGAAAAAGACAACTCTGGC
10CpG_qPCR_F	GAACTCACACACAACACCA
10CpG_qPCR_R	ACTCTGAATACCGACTCAAT
15CpG_qPCR_F	TATCACTGTTGATTCTCGC
15CpG_qPCR_R	GGTAAAGAGTTTGGATTAGG
