# Supplementary material for: Neurofibromin 1 controls metabolic balance and Notch-dependent quiescence of murine juvenile myogenic progenitors
Source: Nat Commun. 2024 Feb 15;15:1393. doi: 10.1038/s41467-024-45618-z (PMC10869796; doi:10.1038/s41467-024-45618-z)
Supplement: Supplementary file 3 — Description of additional supplementary files [file 41467_2024_45618_MOESM3_ESM.pdf]

DESCRIPTION OF ADDITIONAL SUPPLEMENTARY FILES

Supplementary Data 1: Raw RPKM values and DESeq2 analysis of transcriptome data from FACS-isolated p7 control and Nf1Myf5 myogenic progenitors.

Supplementary Data 2: custom gene list for muscle stem cell quiescence- and activation associated genes, and their representation in the transcriptome of p7 control and Nf1Myf5 MPs.

Supplementary Data 3: List of genes with significantly decreased H3K27me3 levels in Nf1Myf5 vs. control p7 MPs.

Supplementary Data 4: List of genes with significantly decreased H3K27me3 levels and downregulated mRNA abundance in Nf1Myf5 vs. control p7 MPs.
