# Supplementary material for: Neurofibromin 1 controls metabolic balance and Notch-dependent quiescence of murine juvenile myogenic progenitors
Source: Nat Commun. 2024 Feb 15;15:1393. doi: 10.1038/s41467-024-45618-z (PMC10869796; doi:10.1038/s41467-024-45618-z)
Supplement: Supplementary file 8 — Reporting Summary [file 41467_2024_45618_MOESM8_ESM.pdf]

nature portfolio

Corresponding author(s): Sigmar Stricker

Last updated by author(s): Jan 24, 2024

Reporting Summary

Nature Portfolio wishes to improve the reproducibility of the work that we publish. This form provides structure for consistency and transparency in reporting. For further information on Nature Portfolio policies, see our Editorial Policies and the Editorial Policy Checklist.

Statistics

For all statistical analyses, confirm that the following items are present in the figure legend, table legend, main text, or Methods section.

n/a	Confirmed	
☐	☒	The exact sample size (*n*) for each experimental group/condition, given as a discrete number and unit of measurement
☐	☒	A statement on whether measurements were taken from distinct samples or whether the same sample was measured repeatedly
☐	☒	The statistical test(s) used AND whether they are one- or two-sided<br>*Only common tests should be described solely by name; describe more complex techniques in the Methods section.*
☐	☒	A description of all covariates tested
☐	☒	A description of any assumptions or corrections, such as tests of normality and adjustment for multiple comparisons
☐	☒	A full description of the statistical parameters including central tendency (e.g. means) or other basic estimates (e.g. regression coefficient) AND variation (e.g. standard deviation) or associated estimates of uncertainty (e.g. confidence intervals)
☐	☒	For null hypothesis testing, the test statistic (e.g. *F*, *t*, *r*) with confidence intervals, effect sizes, degrees of freedom and *P* value noted<br>*Give P values as exact values whenever suitable.*
☒	☐	For Bayesian analysis, information on the choice of priors and Markov chain Monte Carlo settings
☒	☐	For hierarchical and complex designs, identification of the appropriate level for tests and full reporting of outcomes
☒	☐	Estimates of effect sizes (e.g. Cohen's *d*, Pearson's *r*), indicating how they were calculated

Our web collection on statistics for biologists contains articles on many of the points above.

Software and code

Policy information about availability of computer code

Data collection	Zeiss ZEN 3.6; Leica LAS X
Data analysis	ImageJ; Seahorse Wave Desktop Software; SDS software version 2.4; STAR 2.4.2a software with mouse genome (mm9); DESeq2; GSEA software 4.0.1 desktop; DAVID 6.8; bwa v0.7.15; samtools rmdup; QSEA Bioconductor package v.1.12.0;

For manuscripts utilizing custom algorithms or software that are central to the research but not yet described in published literature, software must be made available to editors and reviewers. We strongly encourage code deposition in a community repository (e.g. GitHub). See the Nature Portfolio guidelines for submitting code & software for further information.

Data

Policy information about availability of data

All manuscripts must include a data availability statement. This statement should provide the following information, where applicable:

- Accession codes, unique identifiers, or web links for publicly available datasets
- A description of any restrictions on data availability
- For clinical datasets or third party data, please ensure that the statement adheres to our policy

The sequencing raw data generated in this study have been deposited in the Gene Expression Omnibus (GEO) database under the Super Series accession number GSE159026. All other data generated in this study are provided in the Supplementary Information/Source Data file.

Research involving human participants, their data, or biological material

Policy information about studies with human participants or human data. See also policy information about sex, gender (identity/presentation), and sexual orientation and race, ethnicity and racism.

Reporting on sex and gender	No human participants, data, or biological material was used
Reporting on race, ethnicity, or other socially relevant groupings	No human participants, data, or biological material was used
Population characteristics	No human participants, data, or biological material was used
Recruitment	No human participants, data, or biological material was used
Ethics oversight	No human participants, data, or biological material was used

Note that full information on the approval of the study protocol must also be provided in the manuscript.

Field-specific reporting

Please select the one below that is the best fit for your research. If you are not sure, read the appropriate sections before making your selection.

☒ Life sciences ☐ Behavioural & social sciences ☐ Ecological, evolutionary & environmental sciences

For a reference copy of the document with all sections, see nature.com/documents/nr-reporting-summary-flat.pdf

Life sciences study design

All studies must disclose on these points even when the disclosure is negative.

Sample size	Sample sizes are clearly reported in the figure legend. For results presented in Figs 1-6, S1-S8, no sample size calculation was performed, as there was no expected effect size. Sample numbers were chosen based on previous experience with the Nf1maf5 Cre mutant line (Reference 31) and based on the limited number of mutant mice available. Statistical analysis was performed after after data collection. For DAPT treatment (Fig. 7, S9), power analysis was performed before the experiment to determine the minimum number of necessary biological replicates to observe a minimum effect size of 10% Pax7 cell increase in the DAPT vs the placebo group.
Data exclusions	No data were excluded.
Replication	A minimum of 3 biological replicates was used for all experiments with exception of RNA Sequencing. For cost reasons 2 biological replicates were used in agreement with ENCODE guidelines. In vitro experiments were performed 3 times using independent biological samples (primary cells).
Randomization	Mice were allocated to groups based on genotype. Male and female mice were randomly assigned into the experimental groups.
Blinding	Investigators were not blinded during data collection and analysis. For financial reasons, mouse genotyping, sample preparation and analysis was performed by the same person.

Reporting for specific materials, systems and methods

We require information from authors about some types of materials, experimental systems and methods used in many studies. Here, indicate whether each material, system or method listed is relevant to your study. If you are not sure if a list item applies to your research, read the appropriate section before selecting a response.

Materials & experimental systems

n/a	Involved in the study	
☐	☒	Antibodies
☒	☐	Eukaryotic cell lines
☒	☐	Palaeontology and archaeology
☐	☒	Animals and other organisms
☒	☐	Clinical data
☒	☐	Dual use research of concern
☒	☐	Plants

Methods

n/a	Involved in the study	
☐	☒	ChIP-seq
☒	☐	Flow cytometry
☒	☐	MRI-based neuroimaging

Antibodies

Antibodies used

Commercial antibodies:
Goat anti-Collagen IV; Millipore Cat# AB769; RRID: AB_92262
Goat anti-Desmin; R&D Systems Cat# AF3844; RRID: AB_2092419
Mouse anti-Pax7; DSHB Cat# pax7, RRID: AB_528428
Rabbit anti-Ki67; Abcam Cat# ab16667, RRID: AB_302459
Mouse anti-Ki67; BD Biosciences Cat# 550609, clone B56; RRID: AB_393778
Rabbit anti-MyoD; Cell Signaling Technology Cat# 13812, clone D8G3; RRID: AB_2798320
Mouse anti-MyoD; BD Biosciences Cat# 554130, clone 5.8A; RRID: AB_395255
Mouse anti-MF20; DSHB Cat# MF 20, RRID: AB_2147781
Anti-Myosin (Skeletal, Fast) antibody; Sigma-Aldrich Cat# M1570, clone MY-32; RRID: AB_ 2147168
Mouse anti-MyHC type 1; DSHB Cat# BA-D5, RRID: AB_2235587
Mouse anti-MyHC type 2A; DSHB Cat# SC-71, RRID: AB_2147165
Mouse anti-MyHC type 2B; DSHB Cat# BF-F3, RRID: AB_2266724
Rabbit anti-phospho (Thr 389)-p70s6k; Cell Signaling Technology Cat# 9205, RRID: AB_330944
Rabbit anti-p70s6k; Cell Signaling Technology Cat# 9202, RRID: AB_331676
Rabbit anti-pERK1/2; Cell Signaling Technology Cat# 9101, RRID: AB_331646
Rabbit anti-Phospho-S6 Ribosomal Protein (Ser235/236); Cell Signaling Technology Cat# 4858, clone D57.2.2E; RRID: AB_916156
Anti-acetyl-Histone H4 (Lys16); Millipore Cat# 07-329, RRID: AB_310525
Anti-trimethyl-Histone H3 (Lys4); Millipore Cat# 07-473, RRID: AB_1977252
Anti-trimethyl-Histone H3 (Lys27); Millipore Cat# 07-449, RRID: AB_310624
Mouse anti-β-Tubulin III; Sigma-Aldrich Cat# T8578, clone 2G10; RRID: AB_1841228
Alexa Fluor 488 Donkey anti-Mouse; Thermo Fisher Cat# A-21202, RRID: AB_141607
Alexa Fluor 568 Donkey anti-Mouse; Thermo Fisher Cat# A10037, RRID: AB_2534013
Alexa Fluor 488 Donkey anti-Rabbit; Thermo Fisher Cat# A-21206, RRID: AB_141708
Alexa Fluor 568 Donkey anti-Rabbit; Thermo Fisher Cat# A10042, RRID: AB_2534017
Alexa Fluor 488 Donkey anti-Goat; Thermo Fisher Cat# A-11055, RRID: AB_2534102
Alexa Fluor 568 Donkey anti-Goat; Thermo Fisher Cat# A-11057, RRID: AB_2534104
Alexa Fluor 680 Donkey anti-Goat; Thermo Fisher Cat# A-21084, RRID: AB_2535741
Alexa Fluor 680 Goat anti-Guinea pig; Thermo Fisher Cat# SA5-10098, RRID: AB_2556678
Alexa Fluor 647 Goat anti-Mouse IgG1; Thermo Fisher Cat# A-21240, RRID: AB_2535809
Alexa Fluor 488 Goat anti-Mouse IgM (Heavy chain); Thermo Fisher Cat# A-21042, RRID: AB_2535711
Alexa Fluor 555 Goat anti-Mouse IgG2b; Thermo Fisher Cat# A-21147, RRID: AB_2535783
HRP Goat anti-Rabbit; Thermo Fisher Cat# A27036, RRID: AB_2536099
HRP Goat anti-Mouse; Thermo Fisher Cat# G-21040, RRID: AB_2536527
Rat anti-CD31(PECAM1) APC; Thermo Fisher Cat# 17-0311-82, clone 390; RRID: AB_657735
Rat anti-CD45 APC; Thermo Fisher Cat# 17-0451-83, clone 30-F11; RRID: AB_469393
Rat anti-Ter119 APC; Thermo Fisher Cat# 17-5921-83, clone TER-119; RRID: AB_469474
Rat anti-Ly-6A/E (Sca1) APC-Cy7; BioLegend Cat# 108126, clone D7; RRID: AB_10645327
Rat anti-Integrin alpha 7 PE; R&D Systems Cat# FAB3518P, clone 334908; RRID: AB_2128441

Gifted antibodies:
Guinea pig anti-Pax7; Carmen Birchmeier, Max Delbrück Center for Molecular Medicine Berlin, cbirch@mdc-berlin.de

Validation

Commercial antibodies:
validation available on the company website
Goat anti-Collagen IV https://www.merckmillipore.com/DE/de/product/Anti-Collagen-Type-IV-Antibody,MM_NF-AB769?ReferrerURL=https%3A%2F%2Fwww.google.com%2F
Goat anti-Desmin https://www.rndsystems.com/products/human-mouse-desmin-antibody_af3844
Mouse anti-Pax7 https://dshb.biology.uiowa.edu/PAX7
Rabbit anti-Ki67 https://www.abcam.com/en-at/products/primary-antibodies/ki67-antibody-sp6-ab16667
Mouse anti-Ki67 https://www.bdbiosciences.com/en-in/products/reagents/flow-cytometry-reagents/research-reagents/single-color-antibodies-ruo/purified-mouse-anti-ki-67.550609
Rabbit anti-MyoD https://www.cellsignal.com/products/primary-antibodies/myod1-d8g3-xp-rabbit-mab/13812
Mouse anti-MyoD https://www.bdbiosciences.com/en-us/products/reagents/microscopy-imaging-reagents/immunohistochemistry-reagents/purified-mouse-anti-myod.554130
Mouse anti-MF20 https://dshb.biology.uiowa.edu/MF-20
Anti-Myosin (Skeletal, Fast) antibody https://www.sigmaaldrich.com/DE/de/product/sigma/m1570
Mouse anti-MyHC type 1 https://dshb.biology.uiowa.edu/BA-D5
Mouse anti-MyHC type 2A https://dshb.biology.uiowa.edu/SC-71
Mouse anti-MyHC type 2B https://dshb.biology.uiowa.edu/BF-F3
Rabbit anti-phospho (Thr 389)-p70s6k https://www.cellsignal.com/products/primary-antibodies/phospho-p70-s6-kinase-thr389-antibody/9205
Rabbit anti-p70s6k https://www.cellsignal.com/products/primary-antibodies/p70-s6-kinase-antibody/9202
Rabbit anti- pERK1/2 https://www.cellsignal.com/products/primary-antibodies/phospho-p44-42-mapk-erk1-2-thr202-tyr204-antibody/9101
Rabbit anti-Phospho-S6 Ribosomal Protein (Ser235/236) https://www.cellsignal.com/products/primary-antibodies/phospho-s6-ribosomal-protein-ser235-236-d57-2-2e-xp-rabbit-mab/4858
Anti-acetyl-Histone H4 (Lys16) https://www.merckmillipore.com/DE/de/product/Anti-acetyl-Histone-H4-Lys16-Antibody,MM_NF-07-329
Anti-trimethyl-Histone H3 (Lys4) https://www.merckmillipore.com/DE/de/product/Anti-trimethyl-Histone-H3-Lys4-Antibody,MM_NF-07-473
Anti-trimethyl-Histone H3 (Lys27) https://www.merckmillipore.com/DE/de/product/Anti-trimethyl-Histone-H3-Lys27-

Antibody,MM_NF-07-449
Mouse anti-β-Tubulin III https://www.sigmaaldrich.com/DE/de/product/sigma/t8578
Alexa Fluor 488 Donkey anti-Mouse https://www.thermofisher.com/antibody/product/Donkey-anti-Mouse-IgG-H-L-Highly-Cross-Adsorbed-Secondary-Antibody-Polyclonal/A-21202
Alexa Fluor 568 Donkey anti-Mouse https://www.thermofisher.com/antibody/product/Donkey-anti-Mouse-IgG-H-L-Highly-Cross-Adsorbed-Secondary-Antibody-Polyclonal/A10037
Alexa Fluor 488 Donkey anti-Rabbit https://www.thermofisher.com/antibody/product/Donkey-anti-Rabbit-IgG-H-L-Highly-Cross-Adsorbed-Secondary-Antibody-Polyclonal/A-21206
Alexa Fluor 568 Donkey anti-Rabbit https://www.thermofisher.com/antibody/product/Donkey-anti-Rabbit-IgG-H-L-Highly-Cross-Adsorbed-Secondary-Antibody-Polyclonal/A10042
Alexa Fluor 488 Donkey anti-Goat https://www.thermofisher.com/antibody/product/Donkey-anti-Goat-IgG-H-L-Cross-Adsorbed-Secondary-Antibody-Polyclonal/A-11055
Alexa Fluor 568 Donkey anti-Goat https://www.thermofisher.com/antibody/product/Donkey-anti-Goat-IgG-H-L-Cross-Adsorbed-Secondary-Antibody-Polyclonal/A-11057
Alexa Fluor 680 Donkey anti-Goat https://www.thermofisher.com/antibody/product/Donkey-anti-Goat-IgG-H-L-Cross-Adsorbed-Secondary-Antibody-Polyclonal/A-21084
Alexa Fluor 680 Goat anti-Guinea pig https://www.thermofisher.com/antibody/product/Goat-anti-Guinea-Pig-IgG-H-L-Cross-Adsorbed-Secondary-Antibody-Polyclonal/SA5-10098
Alexa Fluor 647 Goat anti-Mouse IgG1 https://www.thermofisher.com/antibody/product/Goat-anti-Mouse-IgG1-Cross-Adsorbed-Secondary-Antibody-Polyclonal/A-21240
Alexa Fluor 488 Goat anti-Mouse IgM (Heavy chain) https://www.thermofisher.com/antibody/product/Goat-anti-Mouse-IgM-Heavy-chain-Cross-Adsorbed-Secondary-Antibody-Polyclonal/A-21042
Alexa Fluor 555 Goat anti-Mouse IgG2b https://www.thermofisher.com/antibody/product/Goat-anti-Mouse-IgG2b-Cross-Adsorbed-Secondary-Antibody-Polyclonal/A-21147
HRP Goat anti-Rabbit https://www.thermofisher.com/antibody/product/Goat-anti-Rabbit-IgG-Heavy-chain-Secondary-Antibody-Recombinant-Polyclonal/A27036
HRP Goat anti-Mouse https://www.thermofisher.com/antibody/product/Goat-anti-Mouse-IgG-H-L-Cross-Adsorbed-Secondary-Antibody-Polyclonal/G-21040
Rat anti-CD31(PECAM1) APC https://www.thermofisher.com/antibody/product/CD31-PECAM-1-Antibody-clone-390-Monoclonal/17-0311-82
Rat anti-CD45 https://www.thermofisher.com/antibody/product/CD45-Antibody-clone-30-F11-Monoclonal/17-0451-82
Rat anti-Ter119 APC https://www.thermofisher.com/antibody/product/TER-119-Antibody-clone-TER-119-Monoclonal/17-5921-82
Rat anti-Ly-6A/E (Sca1) APC-Cy7 https://www.biolegend.com/en-us/products/apc-cyanine7-anti-mouse-ly-6a-e-sca-1-antibody-6752
Rat anti-Integrin alpha 7 PE https://www.rndsystems.com/products/mouse-integrin-alpha7-pe-conjugated-antibody-334908_fab3518p

Gifted antibodies:
Guinea pig anti-Pax7 was tested on Pax7 mutant tissue (see Zhang et al. 2021 Nature Communications, https://doi.org/10.1038/s41467-021-21631-4)

Animals and other research organisms

Policy information about studies involving animals; ARRIVE guidelines recommended for reporting animal research, and Sex and Gender in Research

Laboratory animals

Mouse; Cre-expressing mouse lines: Myf5-Cre: B6.129S4-Myf5tm3(cre)Sor/J, Acta1-Cre: B6.Cg-Tg(ACTA1-cre)79Jme/J were crossed to Nf1 floxed mice: Nf1tm1Par/J, to generate Nf1Myf5 and Nf1Acta1 animals. Animals were analyzed between 7 days and 15 weeks of age. To assess Cre specificity, Cre lines were crosed with mTmG reporter mice: Gt(ROSA)26Sortm4(ACTB-tdTomato,-EGFP)Luo/J.

Myf5Cre and Acta1Cre mice were obtained from Carmen Birchmeier (Max Delbrück Center for Molecular Medicine Berlin, Germany, cbirch@mdc-berlin.de)
Nf1tm1Par/J mice were obtained from the Jackson Laboratory
Gt(ROSA)26Sortm4(ACTB-tdTomato,-EGFP)Luo/J mice were were obtained from Andreas Kispert (Medizinische Hochschule Hannover, Germany, kispert.andreas@mh-hannover.de)

Wild animals

No wild animals were used in this study

Reporting on sex

Male and female mice were randomly assigned into the experimental groups, sex-based analysis was not performed due to low animal numbers.

Field-collected samples

No field collected samples were used in this study

Ethics oversight

Animal maintenance and experiments were approved by the "Landesamt für Gesundheit und Soziales" Berlin, Germany (ZH120, G0346/13, G0176/19, G0270/18).

Note that full information on the approval of the study protocol must also be provided in the manuscript.

Plants

Seed stocks

Report on the source of all seed stocks or other plant material used. If applicable, state the seed stock centre and catalogue number. If plant specimens were collected from the field, describe the collection location, date and sampling procedures.

Novel plant genotypes

Describe the methods by which all novel plant genotypes were produced. This includes those generated by transgenic approaches,

Novel plant genotypes	*gene editing, chemical/radiation-based mutagenesis and hybridization. For transgenic lines, describe the transformation method, the number of independent lines analyzed and the generation upon which experiments were performed. For gene-edited lines, describe the editor used, the endogenous sequence targeted for editing, the targeting guide RNA sequence (if applicable) and how the editor was applied.*
Authentication	*Describe any authentication procedures for each seed stock used or novel genotype generated. Describe any experiments used to assess the effect of a mutation and, where applicable, how potential secondary effects (e.g. second site T-DNA insertions, mosiacism, off-target gene editing) were examined.*

ChIP-seq

Data deposition

☒ Confirm that both raw and final processed data have been deposited in a public database such as GEO.

☒ Confirm that you have deposited or provided access to graph files (e.g. BED files) for the called peaks.

Data access links<br>*May remain private before publication.*	Gene Expression Omnibus (GEO) Super Series accession number GSE159026
Files in database submission	bigwig files/ peak text files
Genome browser session<br>(e.g. UCSC)	https://genome.ucsc.edu/s/xiaoyan%20wei/MeDIP_Chipseq

Methodology

Replicates	2 biological replicates were used
Sequencing depth	Illumina 2 x 75 base paired-end
Antibodies	Antibodies are listed in Supplementary Table 1
Peak calling parameters	MACS2 version 2.1.2;effective genome size = 1.87e+09;band width = 300;model fold = [5, 50]; qvalue cutoff for narrow/strong regions = 5.00e-02;Range for calculating regional lambda is: 10000 bps.
Data quality	At least 1685 peaks have been identified with FDR 5% and above 5-fold enrichment.
Software	MACS2; R studio
